# Interventricular Differences of Signaling Pathways-Mediated Regulation of Cardiomyocyte Function in Response to High Oxidative Stress in the Post-Ischemic Failing Rat Heart

**DOI:** 10.3390/antiox10060964

**Published:** 2021-06-16

**Authors:** Árpád Kovács, Melissa Herwig, Heidi Budde, Simin Delalat, Detmar Kolijn, Beáta Bódi, Roua Hassoun, Melina Tangos, Saltanat Zhazykbayeva, Ágnes Balogh, Dániel Czuriga, Sophie Van Linthout, Carsten Tschöpe, Naranjan S. Dhalla, Andreas Mügge, Attila Tóth, Zoltán Papp, Judit Barta, Nazha Hamdani

**Affiliations:** 1 Division of Clinical Physiology, Faculty of Medicine, University of Debrecen, 4032 Debrecen, Hungary; kovacs.arpad@med.unideb.hu (Á.K.); bodibea0509@gmail.com (B.B.); atitoth@med.unideb.hu (A.T.); pappz@med.unideb.hu (Z.P.); 2 Institut für Forschung und Lehre (IFL) Molecular and Experimental Cardiology, St. Josef-Hospital, Ruhr University Bochum, 44801 Bochum, Germany; melissa.herwig@rub.de (M.H.); Heidi.Budde@ruhr-uni-bochum.de (H.B.); Simin.Delalat@ruhr-uni-bochum.de (S.D.); Detmar.kolijn@rub.de (D.K.); Roua.Hassoun@ruhr-uni-bochum.de (R.H.); melli-sw@web.de (M.T.); Saltanat.Zhazykbayeva@ruhr-uni-bochum.de (S.Z.); Andreas.Muegge@ruhr-uni-bochum.de (A.M.); 3 Department of Cardiology, St. Josef-Hospital, Ruhr University Bochum, 44801 Bochum, Germany; 4 Department of Cardiology, Faculty of Medicine, University of Debrecen, 4032 Debrecen, Hungary; agnesbalogh@med.unideb.hu (Á.B.); dczuriga@med.unideb.hu (D.C.); bartajud@gmail.com (J.B.); 5 Berlin Institute of Health at Charite (BIH)-Universitätmedizin Berlin, BIH Center for Regenerative Therapies (BCRT), 13353 Berlin, Germany; sophie.van-linthout@charite.de (S.V.L.); carsten.tschoepe@charite.de (C.T.); 6 Institute of Cardiovascular Sciences, St. Boniface Hospital Albrechtsen Research Centre, 351 Tache Avenue, Department of Physiology and Pathophysiology, College of Medicine, Faculty of Health Sciences, University of Manitoba, Winnipeg, MB R2H 2A6, Canada; nsdhalla@sbrc.ca; 7 HAS-UD Vascular Biology and Myocardial Pathophysiology Research Group, Hungarian Academy of Sciences, H-4032 Debrecen, Hungary

**Keywords:** oxidative stress, right ventricle, heart failure, HFrEF, diastolic dysfunction, CaMKII, PKG, cardiomyocyte, passive stiffness, titin, Ca^2+^-sensitivity, myofilament proteins

## Abstract

Standard heart failure (HF) therapies have failed to improve cardiac function or survival in HF patients with right ventricular (RV) dysfunction suggesting a divergence in the molecular mechanisms of RV vs. left ventricular (LV) failure. Here we aimed to investigate interventricular differences in sarcomeric regulation and function in experimental myocardial infarction (MI)-induced HF with reduced LV ejection fraction (HFrEF). MI was induced by LAD ligation in Sprague–Dawley male rats. Sham-operated animals served as controls. Eight weeks after intervention, post-ischemic HFrEF and Sham animals were euthanized. Heart tissue samples were deep-frozen stored (*n* = 3–5 heart/group) for ELISA, kinase activity assays, passive stiffness and Ca^2+^-sensitivity measurements on isolated cardiomyocytes, phospho-specific Western blot, and PAGE of contractile proteins, as well as for collagen gene expressions. Markers of oxidative stress and inflammation showed interventricular differences in post-ischemic rats: TGF-β1, lipid peroxidation, and 3-nitrotyrosine levels were higher in the LV than RV, while hydrogen peroxide, VCAM-1, TNFα, and TGF-β1 were increased in both ventricles. In addition, nitric oxide (NO) level was significantly decreased, while FN-1 level was significantly increased only in the LV, but both were unchanged in RV. CaMKII activity showed an 81.6% increase in the LV, in contrast to a 38.6% decrease in the RV of HFrEF rats. Cardiomyocyte passive stiffness was higher in the HFrEF compared to the Sham group as evident from significantly steeper F_passive_ vs. sarcomere length relationships. In vitro treatment with CaMKIIδ, however, restored cardiomyocyte passive stiffness only in the HFrEF RV, but had no effect in the HFrEF LV. PKG activity was lower in both ventricles in the HFrEF compared to the Sham group. In vitro PKG administration decreased HFrEF cardiomyocyte passive stiffness; however, the effect was more pronounced in the HFrEF LV than HFrEF RV. In line with this, we observed distinct changes of titin site-specific phosphorylation in the RV vs. LV of post-ischemic rats, which may explain divergent cardiomyocyte stiffness modulation observed. Finally, Ca^2+^-sensitivity of RV cardiomyocytes was unchanged, while LV cardiomyocytes showed increased Ca^2+^-sensitivity in the HFrEF group. This could be explained by decreased Ser-282 phosphorylation of cMyBP-C by 44.5% in the RV, but without any alteration in the LV, while Ser-23/24 phosphorylation of cTnI was decreased in both ventricles in the HFrEF vs. the Sham group. Our data pointed to distinct signaling pathways-mediated phosphorylations of sarcomeric proteins for the RV and LV of the post-ischemic failing rat heart. These results implicate divergent responses for oxidative stress and open a new avenue in targeting the RV independently of the LV.

## 1. Introduction

Right ventricular (RV) function is an independent prognostic factor and determinate of survival in patients with chronic heart failure (HF). Hence, RV dysfunction cardinally influences symptoms and predicts poor prognosis in HF patients with reduced left ventricular (LV) ejection fraction (EF), i.e., HFrEF [1]. Indeed, RV dysfunction is present in the entire HF spectrum for any given degree of pulmonary hypertension, and RV–arterial coupling is prognostically important in HF, both regardless of LV EF [2]. Whereas RV dysfunction is associated with poor clinical outcomes independently of the underlying mechanism of the disease [3], no therapeutic options are available in today’s clinical routine that specifically target the right heart and even diagnostic approaches are limited. Importantly, the LV and RV differ not only in global structure and loading conditions, but also exhibit fundamental differences on the cellular and molecular level, shape, and physiology [4,5,6,7], given that during embryogenesis the RV is derived from a different set of precursor cells than the LV. However, differences between LV and RV are poorly understood and clearly under-recognized [8]. The most common cause of RV dysfunction is chronic left-sided HF [9]. In contrast to the process of LV remodeling, the pathophysiology of RV dysfunction in HFrEF is less understood. Standard HF therapies have failed to improve cardiac function or survival in HF patients with impaired RV function, which suggests a divergence in the molecular mechanisms of RV vs. LV failure [8]. Therefore, improving RV function by medical therapy implies better survival in HFrEF patients with RV dysfunction than those with persistently abnormal or worsening RV function [10].

On the way from asymptomatic RV dysfunction to symptomatic RV failure, RV diastolic dysfunction appears early with impaired RV filling and increased diastolic RV pressures and right atrial pressures, and thereby is associated with an increased risk of nonfatal hospital admissions for HF [11]. RV shows a poor defense capacity against reactive oxygen species (ROS) [12] indicating that RV is sooner and more susceptible to oxidative stress than the LV [13,14], especially during the transition from RV remodeling to overt failure. The primary source of ROS production also shows interventricular differences, suggesting greater mitochondrial ROS generation in RV vs. LV failure [8]. Moreover, the stressed RV promotes and activates pathways of cell death more than the stressed LV [8,15]. Patients with myocardial infarction (MI)-induced cardiac remodeling presents increased RV apoptosis, even when the RV is not involved in myocardial ischemia [15].

Inflammation is associated with failure of both the LV and RV; however, it is unclear whether the RV is at particular risk of maladaptation driven by inflammatory processes. Myocardial inflammation is linked to detrimental processes like cell death, oxidative stress, and maladaptive matrix remodeling. Furthermore, altered cell metabolism and calcium handling is induced by inflammation, and clear evidence proves inflammation to be present in dysfunctional RV [16,17]. Human studies by *Di Salvo* et al. reported interventricular differences of transcriptomes and proteomes in explanted failing hearts [18,19]. RV transcriptome analysis suggests structural changes and abnormalities in inflammatory processes and reveals specific RV vs. LV myocardial biomarkers in HF [18]. In addition, contractile, cytoskeletal, metabolic, signaling, and survival pathways were shown to be different between the RV and the LV, potentially attributable to diverse posttranscriptional regulation in HF [19]. Nothing so far is known about protein kinases, signaling pathways, and titin in RV vs. LV. Titin, spanning the half-sarcomere, is the main determinant of myocardial passive stiffness at the sarcomere level, and its compliance is particularly driven by phosphorylation of numerous phosphosites mediated by various protein kinases [20].

Taken together, in a recent study, we hypothesized interventricular differences of molecular mechanisms leading to diastolic dysfunction under oxidative stress due to HF. For this reason, we elucidated divergent molecular mechanisms that exist between left and right heart remodeling. We investigated protein kinase-mediated cellular diastolic dysfunction in experimental MI-induced HFrEF, in which the initial pathological trigger involves obvious oxidative stress and inflammation in both ventricles. Here we provide new evidence regarding the signaling mechanisms of RV diastolic dysfunction in the post-ischemic failing heart.

## 2. Materials and Methods

### 2.1. Animal Model

Animal experimental procedures were permitted by the Animal Care Committee of the University of Manitoba (protocol #04-005), according to guidelines established by the Canadian Institutes of Health Research. Left ventricular (LV) myocardial infarction (MI) was induced by left anterior descending coronary artery (LAD) ligation in Sprague–Dawley male rats (175–200 g) [21]. Sham-operated animals served as controls, where animals underwent the same procedure as the MI group, except the strangulation around LAD (Sham). Eight weeks after intervention, hemodynamic and general parameters confirmed post-ischemic heart failure (HF) with reduced LV ejection fraction in animals with previous LV MI (HFrEF). Furthermore, HFrEF animals presented an increased right ventricular (RV) weight and lung wet/dry weight ratio [21]. At this time point, animals were euthanized, and their heart tissue samples were deep-frozen stored at −80 °C for subsequent experiments (*n* = 3–5 heart/group). 

### 2.2. Quantification of Tissue Oxidative Stress and Inflammation

Myocardial levels (*n* = 4–5 sample/group) of oxidative stress and inflammatory markers were tested with enzyme-linked immunosorbent assay (ELISA) and colorimetric assay kits: Hydrogen peroxide assay kit (ab102500; Abcam, Cambridge, UK), lipid peroxidation (malondialdehyde) assay kit (ab118970; Abcam), 3-Nitrotyrosine ELISA kit (ab116691; Abcam), nitric oxide (NO) colorimetric assay kit (K262; BioVision, Inc., Milpitas, CA, USA), interleukin-6 (IL-6) ELISA kit (ab100772; Abcam), intercellular cell adhesion molecule-1 (ICAM-1) ELISA kit (ERICAM1; Thermo Fisher Scientific, Waltham, MA, USA), vascular cell adhesion molecule-1 (VCAM-1) ELISA kit (KHT0601; Thermo Fisher Scientific), tumor necrosis factor alpha (TNFα) ELISA kit (ab108913; Abcam), transforming growth factor-beta 1 (TGF-β1) ELISA kit (BMS623-3; Thermo Fisher Scientific), fibronectin 1 (FN-1) ELISA kit (LS-F2425; LifeSpan BioSciences, Seattle, WA, USA).

### 2.3. Force Measurements in Isolated Cardiomyocytes

For mechanical measurements of isolated cardiomyocytes, deep-frozen (−80 °C) RV and LV samples were mechanically disrupted and membrane-permeabilized by Triton X-100 detergent (0.5%; Sigma-Aldrich, St. Louis, MO, USA) for 5 min in relaxing solution (MgCl_2_: 1 mM, KCl: 100 mM, EGTA: 2 mM, ATP: 4 mM, imidazole: 10 mM; pH 7.0) at 4 °C. The cell suspension was washed 5 times in relaxing solution.

Cardiomyocyte (*n* = 8–14 cardiomyocyte/3–5 heart/group) Ca^2+^-independent passive force (F_passive_) was measured in a relaxing buffer at room temperature within a sarcomere length (SL) range between 1.8 and 2.4 μm as described before [22]. Single cardiomyocytes were selected under an inverted microscope (Zeiss Axiovert 135, 40 × objective; Carl Zeiss AG, Oberkochen, Germany) and attached with silicone adhesive between a force transducer and a high-speed length controller (piezoelectric motor) as part of a "Permeabilized Myocyte Test System" (1600A; with force transducer 403A; Aurora Scientific, Aurora, Ontario, Canada). Force values were normalized to the myocyte cross-sectional area calculated from the width and the height of the subjected cell. Following baseline force measurements, cardiomyocytes were incubated for 40 min in a relaxing solution supplemented with either 1) Ca^2+^/calmodulin-dependent protein kinase II (CaMKIIδ; 0.6 µg/mL in the calmodulin-containing buffer; Merck Millipore, Burlington, MA, USA), 2) protein kinase G (PKG1α; 0.1 U/mL, batch 034K1336), cGMP (10 µM) and dithiothreitol (DTT; 6 mM) (all from Sigma-Aldrich, St. Louis, MO, USA), 3) protein kinase A (PKA; 100 U/mL, batch 12K7495; Sigma-Aldrich, St. Louis, MO, USA) and 6 mM DTT (MP Biomedicals, Irvine, CA, USA), or 4) protein kinase Cα (PKCα; 10 μg/mL, batch 93K0330; Sigma-Aldrich, St. Louis, MO, USA) activated in Ca^2+^-containing solution (pCa 5.9) including 6 mM DTT, 1 μM phorbol-12-myristate 13-acetate, and 50 nM calyculin A (all from Sigma-Aldrich, St. Louis, MO, USA). Thereafter, F_passive_ measurements were again performed in relaxing solution at SL 1.8–2.4 μm.

Ca^2+^-sensitivity of skinned cardiomyocytes (*n* = 10–13 cardiomyocyte/3–5 heart/group) was measured as described previously [23]. Cells were attached at each end to a stainless-steel insect needle connecting to either a high-speed length controller (Aurora Scientific) or a sensitive force transducer (SensoNor AS, Horten, Norway) at 15 °C. Subsequent cardiomyocyte isometric force generation was recorded and analyzed at SL of 2.3 μm. Ca^2+^-dependent force production of a single cardiomyocyte was induced by transferring the preparation from relaxing to activating solutions (same compositions as relaxing solution aside from containing CaEGTA instead of EGTA). Ca^2+^-concentrations were indicated as –log_10_[Ca^2+^] values; accordingly, the pCa of relaxing solution was 9, whereas the pCa range of activating solutions was 4.75–7.0. Plots indicating relative active forces of an individual cardiomyocyte at each pCa value were fitted by a specific sigmoidal function in Origin 6.0 analysis program (OriginLab, Northampton, MA, USA). It follows that the pCa value for the half-maximal contraction indicated by pCa_50_ defines per se the Ca^2+^-sensitivity of the contractile machinery. The cross-sectional area was calculated from the width and the height of the subjected cardiomyocytes.

### 2.4. Myocardial CaMKII Activity

CaMKII activity was determined using a CycLex CaMKII assay kit (CY-1173; MBL, Woburn, MA, USA) according to the manufacturer’s protocol. RV and LV tissue samples (*n* = 3 in triplicates/group) were homogenized in sample buffer containing 15% glycerol, 62.5 mM/L Tris (pH 6.8), 1% (*w/v*) SDS, protease inhibitor, and protein phosphatase inhibitor. Homogenates were centrifuged at 10,000 *g* for 15 min at 4 °C. The supernatant was removed and stored at −80 °C. Protein samples were loaded onto microliter wells (~2.0 μg/well) coated with the CaMKII specific substrate, Syntide-2, along with kinase reaction buffer with or without Ca^2+^/calmodulin. To quantify CaMKII activity, a standard curve correlating the amount of active CaMKII and the level of phosphorylation of Syntide-2 was constructed. Accordingly, results of triplicate determinations were averaged and CaMKII activity was expressed as mU/mL.

### 2.5. Myocardial PKG Activity

RV and LV tissues samples (*n* = 3 in triplicates/group) were homogenized in 25 mM Tris-HCl (pH 7.4), 1 mM EDTA, 2 mM EGTA, 5 mM DTT, 0.05% Triton X-100, and protease inhibitor cocktail (all from Sigma-Aldrich, St. Louis, MO, USA) and centrifuged for 5 min. Supernatants containing equal amounts of total protein were analyzed for PKG activity as described previously [24]. Briefly, reaction mixtures were incubated at 30 °C for 10 min. Reaction mixtures contained 40 mM Tris-HCl (pH 7.4), 20 mM Mg(CH_3_COO)_2_, 0.2 mM [^32^P] adenosine triphosphate (ATP) (500–1000 cpm pM–1; Amersham, Little Chalfont, UK), 113 mg/mL heptapeptide (RKRSRAE), and 3 μM cGMP (both from Promega, Madison, WI, USA), and a highly specific inhibitor of cyclic adenosine monophosphate-dependent protein kinase (5–24; Calbiochem, San Diego, CA, USA). The reaction was terminated by spotting 70 μL onto Whatman P-81 filters (MACHEREY-NAGEL). Samples were subsequently incubated and washed with 75 mM H_3_PO_4_ for 5 min to remove unbound ATP. Filters were then washed with 100% ethanol and air dried before quantification. PKG activity was quantified using a Wallac 1409 Liquid Scintillation Counter (Hidex Oy, Turku, Finland). Specific activity of PKG was expressed as pM of ^32^P incorporated into the substrate (pM/min/mg protein). Results of triplicate determinations were averaged.

### 2.6. Myocardial PKA and PKC Activities

PKA and PKC activities were analyzed using a non-radioactive kinase activity assay kit (Enzo Life Sciences, Farmingdale, NY, USA). RV and LV samples (*n* = 3 in triplicates/group) were homogenized in cell lysis buffer (in mM/L: 20 MOPS, 50 β-glycerolphosphate, 50 sodium fluoride, 1 sodium vanadate, 5 EGTA, 2 EDTA, 1 DTT, 1 benzamidine, and 1 PMSF; 1% NP40; 10 μg/mL leupeptin and aprotinin, each). Supernatants were collected after centrifugation at 13,000 rpm for 30 min. Supernatants containing equal amounts of total protein (30 ng/μL protein aliquots were assayed according to manufacturer’s instructions) were added into the appropriate wells of the PKA or PKC substrate microliter plate. Kinase reaction was initiated by addition of ATP, and samples were subsequently incubated for 90 min at 30 °C. Phosphorylated peptide substrates were recognized by phospho-specific substrate antibody. The phospho-specific antibody was subsequently bound by a peroxidase conjugated secondary antibody anti-rabbit IgG:HRP. The assay was developed with tetramethylbenzidine, and the intensity of the color was measured in a microplate reader at 450 nm. Results of triplicate determinations were averaged and specific activity of PKA was expressed as ng/mL and for PKC as µg/µL.

### 2.7. Determination of Myofilament Protein Phosphorylation

We assessed protein phosphorylation with a gel staining-based method [25]. For this, RV and LV tissue samples from Sham and HF animals were prepared as the same as for the mechanical measurements, then were dissolved in sodium dodecyl sulfate (SDS) sample buffer (containing 8 M urea, 2 M thiourea, 3% SDS, 75 mM DTT, 0.05 M Tris-HCL (pH 6.8), 10% glycerol, 0.004% brome-phenol blue, leupeptin: 40 μM, E-64: 10 μM; all from Sigma-Aldrich, St. Louis, MO, USA). Following centrifugation (16,000 *g* for 5 min at 24 °C), the protein amount of supernatant was estimated by the dot-blot technique and was set to 1 mg/mL concentration correlating the sample to bovine serum albumin standards. One dimensional polyacrylamide gel electrophoresis (PAGE) was applied by 2% agarose-strengthened (*n* = 23–47 SDS-PAGE/3–5 heart/group), 4% and 15% (*n* = 8–20 SDS-PAGE/3–5 heart/group) gels to detect titin, cardiac myosin binding protein-C (cMyBP-C), and myofilament proteins below 100 kDa molecular weight, respectively. Gels were run as follows: 2% gel at 2 mA constant current for ~9 h; 4% gel at 30 mA constant current for ~75 min; 15% gel at 30 mA constant current for ~90 min. After running, every gel was stained for 90 min with Pro-Q Diamond (Invitrogen; Molecular Probes, Eugene, OR, USA) phosphoprotein gel stain. Then gels were washed and handled according to the manufacturer’s protocol. Thereafter, gels were stained with Coomassie brilliant blue (Reanal, Budapest, Hungary) protein stain. Protein bands appearing on the subjected gels were documented by MF-ChemiBIS 3.2 gel documentation system (DNR Bio-Imaging Systems, Jerusalem, Israel), identified either necessarily on 2% gels or by a protein standard (ProSieve protein marker from Lonza Rockland, Rockland, ME, USA) on 4% and 15% gels, as their signal intensities were evaluated by ImageJ image processing program (by National Institutes of Health, Bethesda, MD, USA). Accordingly, optical densities shown in arbitrary units (a.u.) in Figure 6B,C were converted to numerical values as area under the curve by MagicPlot Student 2.5.1 analyzing software (Magicplot Systems, Saint Petersburg, Russia). Afterwards, the phosphorylation status of myofilament proteins was determined by the ratio of their phosphorylated form labeled by ProQ Diamond and their own total protein amount labeled by Coomassie blue as described above. Thus, protein phosphorylation in HFrEF was normalized to phosphorylation levels in Sham.

### 2.8. Phospho-Titin Analysis by Western Immunoblot

For titin phosphorylation analysis by Western immunoblot, SDS-PAGE was performed to separate titin as previously described [26]. Briefly, RV and LV tissue samples (*n* = 6–15 sample/3–5 heart/group) were solubilized in 50 mM Tris-SDS buffer (pH 6.8) containing 8 μg/mL leupeptin (Peptide Institute, Ibaraki, Osaka, Japan) and 10 μL/mL phosphatase inhibitor cocktail (P2850; Sigma-Aldrich, St. Louis, MO, USA). Samples were heated for 3 min at 96 °C and centrifuged. Samples were applied in duplicates at concentrations that were within the linear range of the detection system (20 μg and 25 μg dry weight; checked by spectroscopic methods) and separated by agarose-strengthened 1.8% SDS-PAGE. Gels were run at 4 mA constant current for 16 h. Thereafter, Western blot was performed to measure site-specific and total phosphorylation of titin. Following SDS-PAGE, proteins were transferred to polyvinylidene difluoride (PVDF) membranes (Immobilon-P 0.45 µm; Merck Millipore). Blots were pre-incubated with 3% bovine serum albumin in Tween Tris-buffered saline (TTBS; containing: 10 mM Tris-HCl; pH 7.6; 75 mM NaCl; 0.1% Tween; all from Sigma-Aldrich, St. Louis, MO, USA) for 1 h at room temperature. Then, blots were incubated overnight at 4 °C with the 1° antibodies.

An anti-phospho Ser/threonine (Thr) antibody (ECM Biosciences LLC, Versailles, KY, USA; dilution 1:500) was used to assess total titin phosphorylation. Phosphosite-specific anti-titin antibodies were custom-made by Eurogentec (Seraing, Belgium) with positions in N2Bus (N2B unique sequence) and PEVK (rich in proline, glutamate, valine, and lysine amino acids) domains of mouse (*Mus musculus*) titin according to the UniProtKB identifier A2ASS6. The following rabbit polyclonal affinity purified antibodies were used:

- Anti-phospho-N2Bus (Ser-3991) against EEGKS (PO3H2) LSFPLA (dilution 1:500);
- Anti-phospho-N2Bus (Ser-4043) against QELLS (PO3H2) KETLFP (dilution 1:100);
- Anti-phospho-N2Bus (Ser-4080) against LFS (PO3H2) EWLRNI (dilution 1:500);
- Anti-phospho-PEVK (Ser-12742) against EVVLKS (PO3H2) VLRK (dilution 1:100);
- Anti-phospho-PEVK (Ser-12884) against KLRPGS (PO3H2) GGEKPP (dilution 1:500).

The amino acid sequences of rat titin at Ser-3991, Ser-4043, Ser-4080, Ser-12742, and Ser-12884 are identical to the amino acid sequences of mouse, and refer to human titin at Ser-4010, Ser-4062, Ser-4099, Ser-11878, and Ser-12022, respectively [20].

After washing with TTBS, 1° antibody binding was visualized using 2° horseradish peroxidase-labeled (POD), goat anti-rabbit antibody (DakoCytomation, Glostrup, Denmark; dilution 1:10,000), and enhanced chemiluminescence (ECL Western blotting detection; Amersham). Western blot signals were visualized using the LAS-4000 Image Reader and analyzed with Multi Gauge V3.2 software (both from FUJIFILM, Minato, Tokyo, Japan). Coomassie-based PVDF stains were saved for comparison of protein load. Finally, signals obtained from phospho-specific antibodies were normalized to signals obtained from PVDF stains referring to the entire protein amount transferred.

### 2.9. Site-Specific Phosphorylation of Myofilament Proteins

Western immunoblot technique was applied to assess myofilament protein phosphorylation at certain sites. Until the end of running, the same RV and LV tissue samples (*n* = 4–9 sample/3–5 heart/group) from HFrEF and Sham animals were prepared and loaded equally to gel staining methods as detailed above. Unlike that, after separation, proteins from 4% and 15% gels were transferred to nitrocellulose membranes. Membranes were then stained with SYPRO Ruby protein blot stain (Invitrogen; Molecular Probes), washed, and probed appropriately with 1° and 2° antibodies: 1° polyclonal rabbit against phosphorylated serine residue 282 (Ser-282) of cMyBP-C (Enzo Life Sciences; dilution 1:5000), 1° polyclonal rabbit against phosphorylated serine residues 23 and 24 (Ser-23/24) of cardiac Troponin I (cTnI; Abcam; dilution 1:1000), 1° monoclonal mouse against actin (Clone HHF35; DakoCytomation; dilution 1:1000); whereas 2° anti-mouse-POD and anti-rabbit-POD (both from Sigma-Aldrich, St. Louis, MO, USA; dilution 1:40,000) were coupled properly with 1° ones. Protein bands were visualized by enhanced chemiluminescence reaction (ECL), documented by MF-ChemiBIS 3.2 system, and analyzed with ImageJ and MagicPlot Student 2.5.1 software as above. The site-specific phosphorylation level of cMyBP-C at Ser-282 and cTnI at Ser-23/24 in HFrEF was related to the actin expression level from parallel experiments, and thereafter normalized to Sham.

### 2.10. Collagen Gene Expression Analysis

According to established protocols [27], RNA was isolated from frozen RV and LV tissue (*n* = 3/group) using the TRIzol™ method, followed by reverse transcription via the High-Capacity cDNA Reverse Transcription Kit from Applied Biosystems by Thermo Fisher Scientific (Foster City, CA, USA). To determine rat RV and LV Collagen 1a1 (Col1a1) and Collagen 3a1 (Col3a1) mRNA expression, quantitative real-time PCR was performed on a QuantStudio 6 Flex Real-Time PCR System (Thermo Fischer Scientific, Darmstadt, Germany) using the following gene expression assays: Col1a1 (Mm01302043_g1) and Col3a1 (Mm00802331_m1) (all provided by Applied Biosystems, Darmstadt, Germany). For quantification, data were normalized to the endogenous control, CDKN1b (CDKN1b; Mm00438167_g1), and expressed as 2^−∆Ct^.

### 2.11. Data and Statistical Analysis

Group analysis (RV and LV of HFrEF and Sham groups) was done by 2-way ANOVA with Tukey’s multiple comparisons test. For separate analysis, the unpaired t-test was used to compare either HFrEF vs. Sham in the same ventricle, or RV vs. LV in the same group. The effects of in vitro protein kinase incubations (after vs. baseline) and sarcomere lengthening on F_passive_ were compared by the paired t-test of repeated measurements accordingly. Data are given as mean values ± SEM. Statistical significance was assigned when *p* < 0.05.

## 3. Results

### 3.1. Oxidative Stress and Inflammation Show Interventricular Differences in the Post-Ischemic Rat Heart

**Figure 1:**
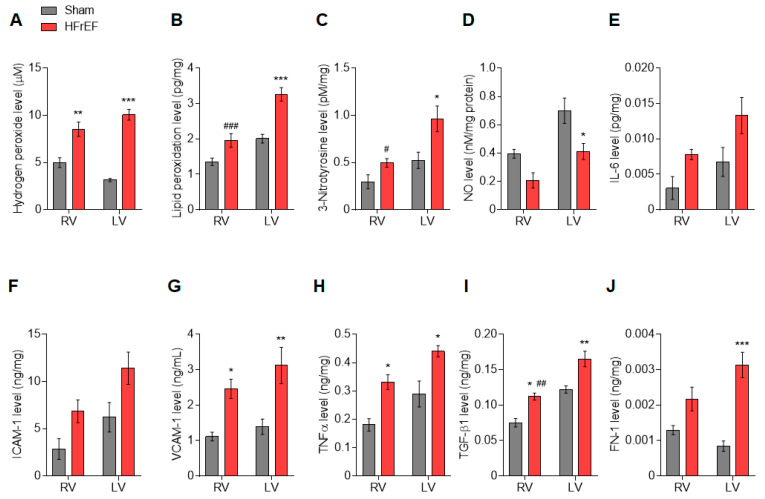
Interventricular differences of oxidative stress and inflammation in the post-ischemic rat heart. Concentration levels of hydrogen peroxide (**A**), lipid peroxidation (**B**), 3-Nitrotyrosine (**C**), nitric oxide (NO; **D**), interleukin 6 (IL-6; **E**), intercellular cell adhesion molecule-1 (ICAM-1; **F**), vascular cell adhesion molecule-1 (VCAM-1; **G**), tumor necrosis factor alpha (TNFα; **H**), transforming growth factor-beta 1 (TGF-β1; **I**), and fibronectin-1 (FN-1; **J**) are shown in the right ventricle (RV) and left ventricle (LV) of post-ischemic (HFrEF) and Sham-operated (Sham) rat hearts. Data are shown as mean ± SEM; *n* = 4–5 sample/group. * *p* < 0.05, ** *p* < 0.01, *** *p* < 0.001 HFrEF vs. Sham (same ventricle); ^#^ *p* < 0.05, ^##^ *p* < 0.01, ^###^ *p* < 0.001 RV vs. LV (same group).

First, we tested myocardial oxidative stress and inflammation in the RV and LV of the post-ischemic heart. A significant increase in the level of hydrogen peroxide in both ventricles indicated a global cardiac oxidative stress after MI (Figure 1A). In contrast, lipid peroxidation (Figure 1B) and 3-Nitrotyrosine (Figure 1C) levels were increased in the LV, but unaltered in the RV of HFrEF animals compared to the Sham group. Similarly, lipid peroxidation (Figure 1B) and 3-Nitrotyrosine (Figure 1C) levels were also higher in the LV vs. RV of HFrEF animals. NO level was decreased (Figure 1D), while FN-1 level was increased (Figure 1J) only in the LV of post-ischemic rats. At the same time, the levels of VCAM-1 (Figure 1G) and TNFα (Figure 1H) were higher in both ventricles in the HFrEF group than those in the Sham group. The level of TGF-β1 was uniformly increased in HFrEF as compared to Sham, but significantly higher in the LV vs. RV of HFrEF animals (Figure 1I). Finally, levels of IL-6 (Figure 1E) and ICAM-1 (Figure 1F) showed a non-significant tendency to be generally higher in HFrEF compared to Sham.

### 3.2. Protein Kinase-Mediated Cardiomyocyte Passive Stiffness Show Interventricular Differences in HFrEF Rats

Next, we investigated main protein kinases of cardiac remodeling that primarily regulate myofilament protein phosphorylation, thereby sarcomere function in the oxidized and inflamed myocardium following MI.

**Figure 2:**
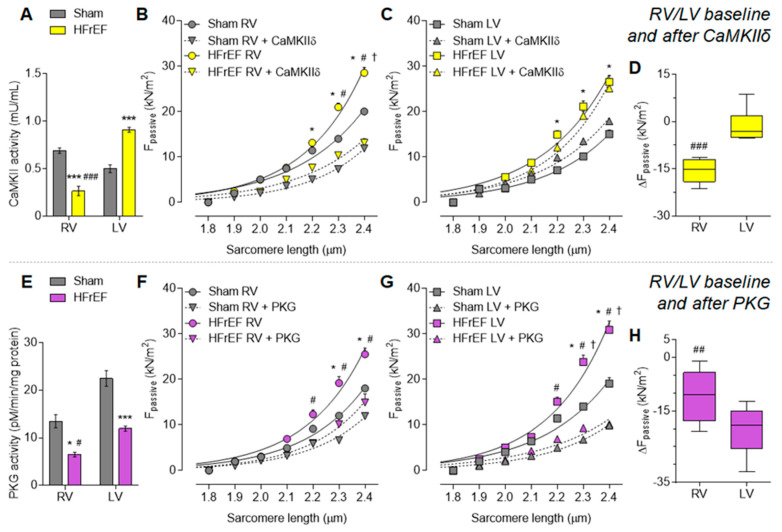
Interventricular differences of protein kinase-mediated cardiomyocyte passive stiffness (F_passive_) in the post-ischemic rat heart: Ca^2+^/calmodulin-dependent protein kinase II (CaMKII) and protein kinase G (PKG). CaMKII activity is shown in HFrEF and Sham groups (**A**). F_passive_ is presented before (baseline) and after CaMKIIδ in vitro administration in the RV (**B**) and the LV (**C**) of HFrEF and Sham hearts, with the corresponding CaMKIIδ in vitro effect (after vs. before) on F_passive_ of HFrEF samples (**D**). PKG activity is shown in HFrEF and Sham groups (**E**). F_passive_ is presented before (baseline) and after PKG in vitro administration in the RV (**F**) and the LV (**G**) of HFrEF and Sham hearts, with the corresponding PKG in vitro effect (after vs. before) on F_passive_ of HFrEF samples (**H**). Data are shown as mean ± SEM; panels A and E: *n* = 3 mean of 3 samples/group; panels B–D and F–H: *n* = 9 cardiomyocyte/3–5 heart/group. Panels A, D–E and H: * *p* < 0.05, *** *p* < 0.001 HFrEF vs. Sham (same ventricle); ^#^ *p* < 0.05, ^##^ *p* < 0.01, ^###^ *p* < 0.001 RV vs. LV (same group). Panels B–C and F–G: * *p* < 0.05 HFrEF vs. Sham; ^#^ *p* < 0.05 HFrEF after CaMKIIδ/PKG vs. HFrEF baseline; ^†^ *p* < 0.05 Sham after CaMKIIδ/PKG vs. Sham.

CaMKII activity was almost doubled (81.6% increase) in the post-ischemic LV vs. Sham LV (Figure 2A). In contrast, CaMKII activity showed a drop in the RV of HFrEF rats (38.6% of the Sham). CaMKII activity was therefore significantly lower in the RV than that of in the LV in post-ischemic hearts (Figure 2A). When testing mechanical changes upon HF remodeling, we observed a general increase in cardiomyocyte passive stiffness in the HFrEF group vs. Sham as evident from significantly steeper F_passive_ vs. sarcomere length relationships both in the RV and LV (Figure 2B,C, respectively). In vitro incubation with CaMKIIδ restored cardiomyocyte passive stiffness only in the HFrEF RV (Figure 2B) but had no effect in the HFrEF LV (Figure 2C), thereby showing a significantly higher passive stiffness lowering effect in the RV vs. LV (Figure 2D) and indicating interventricular differences in CaMKII-mediated functional effects.

PKG activity was generally lower in the failing heart as compared to Sham, both in the RV and LV (Figure 2E). Accordingly, in vitro PKG administration decreased HFrEF cardiomyocyte passive stiffness (Figure 2F,G). Nonetheless, this passive stiffness lowering effect was significantly higher in the LV vs. RV of HFrEF animals (Figure 2H). 

**Figure 3:**
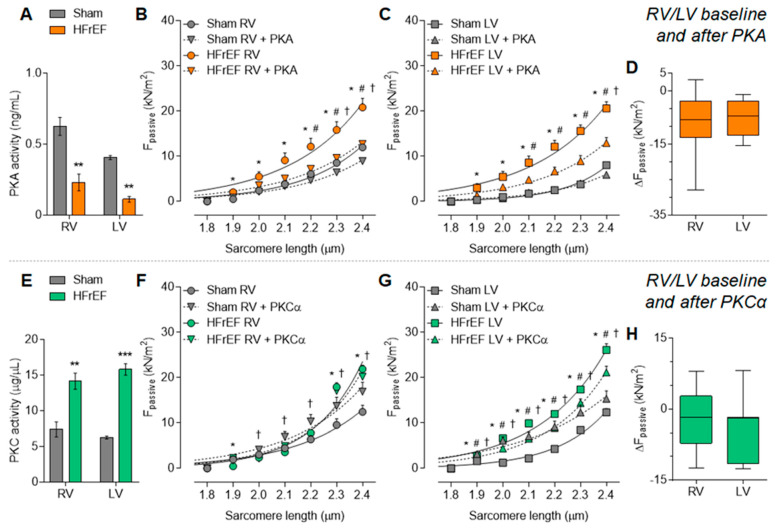
Uniform changes of protein kinase-mediated cardiomyocyte passive stiffness (F_passive_) in the post-ischemic rat heart: Protein kinase A (PKA) and C (PKC). PKA activity is shown in HFrEF and Sham groups (**A**). F_passive_ is presented before (baseline) and after PKA in vitro administration in the RV (**B**) and the LV (**C**) of HFrEF and Sham hearts, with the corresponding PKA in vitro effect (after vs. before) on F_passive_ of HFrEF samples (**D**). PKC activity is shown in HFrEF and Sham groups (**E**). F_passive_ is presented before (baseline) and after PKCα in vitro administration in the RV (**F**) and the LV (**G**) of HFrEF and Sham hearts, with the corresponding PKCα in vitro effect (after vs. before) on F_passive_ of HFrEF samples (**H**). Data are shown as mean ± SEM; panels A and E: *n* = 3 mean of 3 samples/group; panels B–D and F–H: *n* = 8–14 cardiomyocyte/3–5 heart/group. Panels A and E: ** *p* < 0.01, *** *p* < 0.001 HFrEF vs. Sham (same ventricle). Panels B–C and F–G: * *p* < 0.05 HFrEF vs. Sham; ^#^ *p* < 0.05 HFrEF after PKA/PKCα vs. HFrEF baseline; ^†^ *p* < 0.05 Sham after PKA/PKCα vs. Sham.

PKA activity was equally lower in the HFrEF group compared to Sham, both in the RV and LV (Figure 3A). Accordingly, in vitro PKA administration could decrease passive stiffness of both RV and LV cardiomyocytes from HFrEF rats (Figure 3B,C) to the same extent in the two ventricles (Figure 3D).

At the same time, PKC activity was similarly higher in the RV and LV in the HFrEF group as compared to Sham (Figure 3E). In line with the high PKC activity, in vitro PKCα administration had no further effect on passive stiffness of HFrEF cardiomyocytes but increased in the Sham cardiomyocytes (Figure 3F–H).

**Figure 4:**
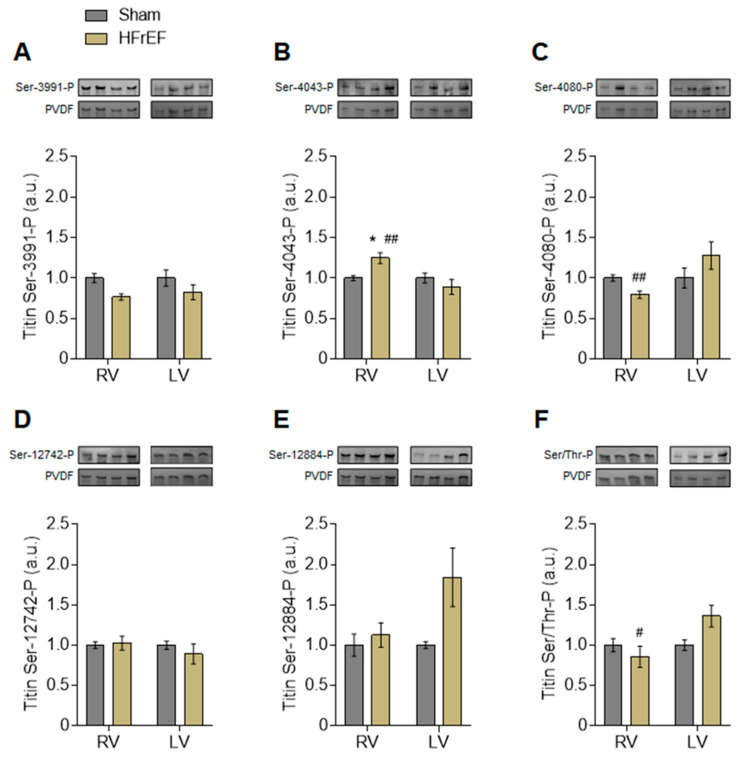
Interventricular differences of titin phosphorylation in the post-ischemic rat heart. Titin phosphorylation (P) levels are shown within the N2Bus domain at serine (Ser)-3991 (**A**), Ser-4043 (**B**) and Ser-4080 (**C**), as well as within the PEVK region at Ser-12742 (**D**) and Ser-12884 (**E**). Phosphorylation at Ser and threonine (Thr) residues is given as total titin phosphorylation (**F**). *Insets*: Representative images show Western immunoblots of duplicates using P-site-specific anti-titin antibodies (top) and corresponding PVDF stains (bottom) at a molecular weight of ~3.0 MDa (titin N2B isoform). Data are shown as mean ± SEM; *n* = 6–15 sample/3–5 heart/group. * *p* < 0.05 HFrEF vs. Sham (same ventricle); ^#^ *p* < 0.05, ^##^ *p* < 0.01 RV vs. LV (same group).

Interventricular differences in protein kinase-mediated mechanical changes in HFrEF led us to investigate the phosphorylation level of titin as the elastic filament of the sarcomere and thereby the main determinant of cardiomyocyte passive stiffness. Based on Coomassie blue protein gel staining, we observed only one clear titin band in each 2% gel referred to as the N2B titin isoform (3.0 MDa; Figure 6A). According to Pro-Q Diamond phosphoprotein gel staining, the total titin phosphorylation was apparently unchanged in the HFrEF group as compared to Sham (Figure 6D). However, site-specific phosphorylation of titin by Western immunoblot showed clear differences between the RV and LV of HFrEF animals (Figure 4A–F). Across the N2Bus of titin, Ser-3991 was similar in the RV vs. LV of HFrEF rats (Figure 4A), while Ser-4043 higher (Figure 4B) and Ser-4080 were lower (Figure 4C) in the RV vs. LV of HFrEF rats. Relative to Sham, Ser-12742 (Figure 4D) and Ser-12884 (Figure 4E) phosphosites of the PEVK domain showed no significant changes in HFrEF, with a strong tendency of increased Ser-12884 phosphorylation in the LV (*p* = 0.07; Figure 4E). Saturation of Ser and Thr residues as total titin phosphorylation was tested by Western immunoblot and showed a significantly lower level in the RV vs. LV of HFrEF animals (Figure 4F). Since titin has numerous phosphosites, these data therefore suggest an extensive redistribution of phosphorylation on titin Ser and Thr residues in the post-ischemic heart.

### 3.3. Myofilament Ca^2+^-Sensitivity and Protein Phosphorylation Show Interventricular Differences in the Failing Rat Heart

**Figure 5:**
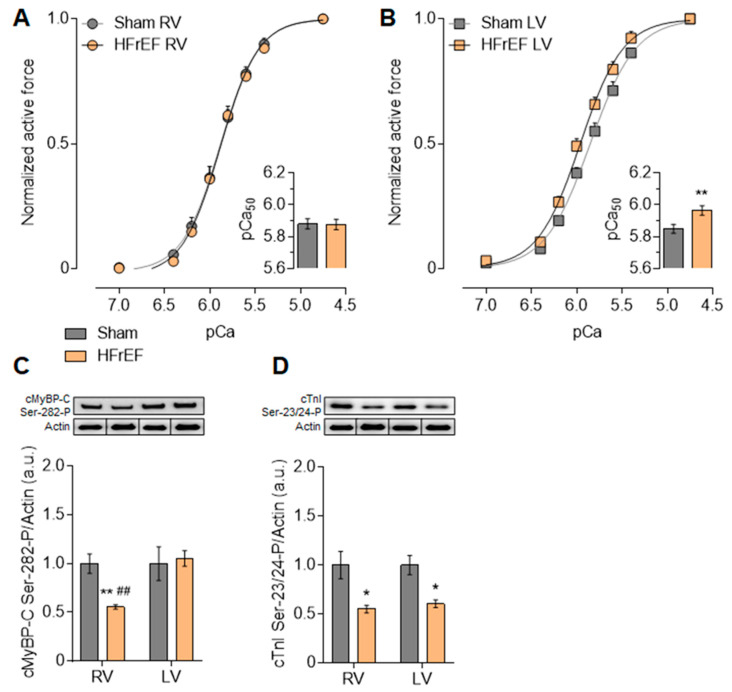
Interventricular differences of myofilament Ca^2+^-sensitivity and protein phosphorylation in the post-ischemic rat heart. Ca^2+^-sensitivities of the contractile machinery of RV (**A**) and LV (**B**) cardiomyocytes are indicated by the pCa_50_ values (*small panels*) derived from normalized active force vs. pCa (–log_10_[Ca^2+^]) relationships. Cardiac myosin binding protein-C (cMyBP-C) phosphorylation at Ser-282 (**C**) and cardiac Troponin I (cTnI) bis-phosphorylation at Ser-23/24 (**D**) are shown after normalization to actin. Insets: Representative images show Western immunoblots using P-site-specific anti-cMyBP-C and anti-cTnI antibodies (top) and corresponding anti-actin antibodies (bottom; discontinuity of the blots indicates identical samples from parallel experiments with different loading order) at molecular weights of ~150 kDa (cMyBP-C), ~24 kDa (cTnI), and ~42 kDa (actin). Data are shown as mean ± SEM; panels A–B: *n* = 10–13 cardiomyocyte/3–5 heart/group; panels C–D: *n* = 4–9 sample/3–5 heart/group. * *p* < 0.05, ** *p* < 0.01 HFrEF vs. Sham (same ventricle); ^##^ *p* < 0.01 RV vs. LV (same group).

Ca^2+^-sensitivity of RV cardiomyocytes did not differ in Sham (5.88 ± 0.03) and HFrEF (5.87 ± 0.03) animals (Figure 5A). Nevertheless, according to the higher pCa_50_ values of LV cardiomyocytes in HFrEF (5.97 ± 0.03) as compared with Sham (5.85 ± 0.03), Ca^2+^-sensitivity of LV cardiomyocytes was increased in the failing heart (Figure 5B).

**Figure 6:**
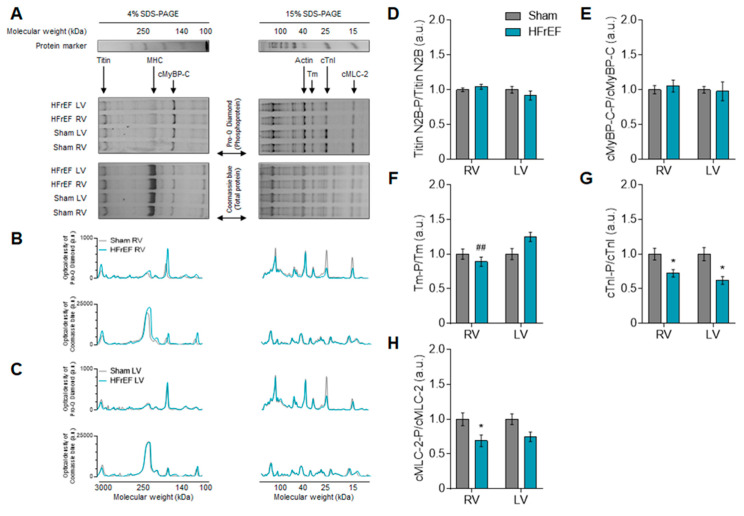
Overall myofilament protein phosphorylation patterns of the RV and the LV in the post-ischemic rat heart. Collection of contractile protein bands in each group is shown by phosphoprotein and protein gel staining of 4% and 15% gels (**A**). SDS-PAGE indicates 1D polyacrylamide gel electrophoresis; MHC, myosin heavy chain; cMyBP-C, cardiac myosin binding protein-C; Tm, tropomyosin; cTnI, cardiac troponin I; MLC-2, myosin light chain 2. Corresponding representations of optical densities of protein gel staining in the RV (**B**) and the LV (**C**) are given in arbitrary units (a.u.). Phosphorylation of titin (**D**), cMyBP-C (**E**), Tm (**F**), cTnI (**G**), and cMLC-2 (**H**) are shown as phosphoprotein/total protein ratios. Data are shown as mean ± SEM; panel D: *n* = 23–47 SDS-PAGE/3–5 heart/group; panels E-H: *n* = 8–20 SDS-PAGE/3–5 heart/group. * *p* < 0.05 HFrEF vs. Sham (same ventricle); ^##^ *p* < 0.01 RV vs. LV (same group).

As myofilament protein phosphorylation levels modulate the Ca^2+^-sensitivity of the contractile machinery due to up- or downregulation of signaling pathways, we therefore investigated small myofilament protein phosphorylation as well. Figure 6A–C demonstrates a comprehensive comparison of protein signal intensities (shown in a.u.) originating from the analysis of single experiments both on 4% and a 15% gels. With similar protein amounts according to Coomassie blue protein gel staining, clear differences appeared in the phosphorylation level of several myofilament proteins by Pro-Q Diamond phosphoprotein gel staining between HFrEF and Sham groups (Figure 6A–C).

Similar to titin, the overall cMyBP-C phosphorylation in HFrEF did not show any differences from Sham in neither ventricle (Figure 6E). However, relative to Sham, site-specific phosphorylation of cMyBP-C at Ser-282 by Western immunoblot was decreased by 44.5% in the RV, but it was not altered in the LV in the HFrEF group (Figure 5C). The significantly lower Ser-282 phosphorylation level of cMyBP-C (Figure 5C) is also in line with the lower CaMKII activity (Figure 2A) in the RV vs. LV in HFrEF rats. In the HFrEF group LV showed a significantly higher Tm phosphorylation in the LV than that of in the RV (Figure 6F). Based on phospho-specific gel staining, cTnI phosphorylation level was equally significantly lower in the RV and in the LV of HFrEF samples relative to Sham (Figure 6G). Accordingly, Ser-23/24 phosphorylation level of cTnI was also decreased in the RV by 45% and in the LV by 39.4% in HFrEF compared to the Sham group (Figure 5D). Relative to Sham, the phosphorylation level of MLC-2 was significantly lower only in the RV, but not in the LV of HFrEF rats (Figure 6H). These data also support diverse protein kinase-mediated myofilament protein phosphorylation in the two ventricles of the failing heart.

### 3.4. RV and LV Structure Show No Differences in Post-Ischemic HFrEF Rats

**Figure 7:**
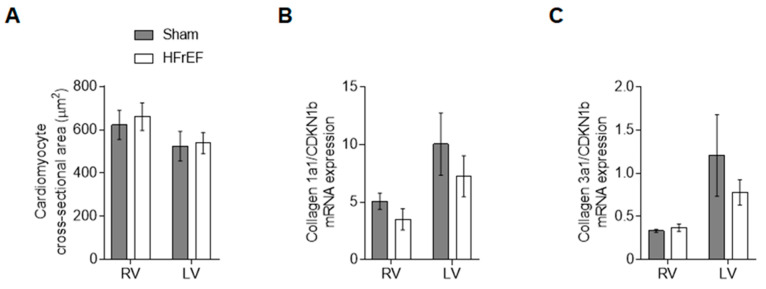
Homogenous ventricular structure in the post-ischemic rat heart. Cardiomyocyte size is given as cross-sectional area (**A**). RV and LV collagen 1a1 (**B**) and collagen 3a1 (**C**) mRNA expression relative to CDKN1b is shown. Data are shown as mean ± SEM; panel A: *n* = 16–27 cardiomyocyte/3–5 heart/group; panels B–C: mean ± SEM of 3 samples/group.

Beyond protein kinase-mediated signaling changes in HFrEF, we investigated myocardium structure related to cardiomyocyte and extracellular matrix as well. Cross-sectional area of the objected RV and LV cells from cellular force measurements (Figure 7A) did not differ in the failing animals (661 ± 63 µm^2^ and 538 ± 49 µm^2^, respectively) as compared to Sham (622 ± 67 µm^2^ and 525 ± 68 µm^2^, respectively). Finally, neither collagen 1a (Figure 7B) nor collagen 3a (Figure 7C) showed differences between HFrEF and Sham groups.

## 4. Discussion

Here we report, for the first time, interventricular differences in protein kinase modulation of cardiomyocyte passive stiffness under diverse oxidative and inflammatory pathways in experimental post-ischemic HFrEF: *1)* CaMKII is downregulated in the RV, while upregulated in the LV; *2)* PKG downregulation shows greater extent in the LV than in the RV. Based on our data, molecular mechanisms of diastolic dysfunction at the sarcomere level likely involve distinctly altered protein kinase signaling pathways in the two ventricles of the failing heart (Table 1).

During the progression of HF, a negative diastolic interaction develops between the two ventricles as reduced EF and dilation of the LV is associated with a parallel stiffening of the RV [28]. Accordingly, under experimental settings, non-occlusive constriction of the left coronary artery of rats resulted in LV failure with increased RV end-diastolic pressure and reduced -dP/dt [29]. Similarly, after LV MI in the mouse, RV dysfunction developed independent of changes in RV afterload [30]. The phenomenon, known as diastolic interdependence of the two ventricles [28], implies not only different hemodynamic stress, but different mechanical and molecular adaptation in the RV vs. LV of the failing heart. Isolated myocardial RV trabeculae from failing human hearts were shown to preserve active force development, while the kinetics of RV relaxation were significantly impaired in an etiology-dependent manner, suggesting even worse RV relaxation kinetics with higher oxidative stress in ischemic compared to non-ischemic hearts [31]. This observation reveals the important role of ROS in the mechanism of diastolic dysfunction in post-ischemic HFrEF [32]. In the recent study, first we extensively characterized RV and LV oxidative stress and inflammation and found a biventricular increased myocardial oxidative and inflammatory status in HFrEF rats following LV MI. Nonetheless, the LV was exposed significantly more to oxidative stress and inflammation than the RV, since NO level was decreased, while lipid peroxidation, 3-Nitrotyrosine, and FN-1 levels increased only in the post-ischemic LV.

The modulation of heart contractility through oxidative stress directly involves the contractile machinery including myofilament proteins. Post-translational modifications of myofilament proteins including oxidation and phosphorylation directly regulate sarcomere function and show a wide range of changes upon cardiac remodeling due to HF [20,33]. In particular, the compliance of titin as the molecular unit of diastolic tension is driven by the phosphorylation pattern of its elastic regions [20], contributing to elevated cardiomyocyte passive tension in human HF [34]. Taking into consideration multiple phosphosites across the elastic regions of the titin molecule, hypo-phosphorylation of the N2Bus and hyper-phosphorylation of the PEVK domain are both involved in the diastolic myocardial stiffness increase in human HFrEF hearts [35]. In the recent study, we observed biventricular increased cardiomyocyte stiffness, but could demonstrate different mechanisms beyond in the RV and LV of post-ischemic HFrEF rats. Despite unchanged total phosphorylation of titin, we found a diverse site-specific phosphorylation pattern of titin, suggesting complex protein-kinase signaling changes in the two ventricles of the failing rat heart.

First of all, CaMKII activity was reduced in the HFrEF RV, while it was increased in the HFrEF LV. As a mechanistic proof of concept, we then demonstrated a significant drop of passive stiffness after in vitro treatment with CaMKIIδ in the RV, thereby restoring phosphorylation of CaMKII-targeted phosphosites on titin. This was in contrast to the LV, in which in vitro CaMKIIδ incubation had no effect on cardiomyocyte stiffness, suggesting already saturated CaMKII-targeted phosphosites on titin. This observation is in clear accordance with our earlier report showing that deranged CaMKII-dependent titin phosphorylation contributes to altered diastolic stress in human HF [36]. However, this is the first time showing interventricular differences in CaMKII-mediated regulation of titin-based passive tension in post-MI HFrEF, suggesting that CaMKII has a differential effect on persistence length of N2Bus between the RV and LV. Interestingly, the blockade of CaMKII promotes ischemic ventricular fibrillation preferentially in the RV of the rabbit heart [37], which also supports the importance of constitutive CaMKII activity in the RV.

On the other hand, we found a global decrease in PKG activity with a larger extent in the LV vs. RV in HFrEF rats. PKG-mediated titin N2Bus phosphorylation is also an important regulator of cardiomyocyte stiffness [20,26], particularly in the failing human heart [24,35]. In our experiments, in vitro PKG incubation corrected passive tension in both ventricles of HFrEF rats, suggesting biventricular PKG-mediated hypophosphorylation of titin, but this functional effect was significantly higher in the LV vs. RV. This observation is clearly in line with the increased oxidative stress and reduced NO level seen in the LV of our HFrEF rats, since comprehensive data support the concept of ROS-induced downregulation of the NO-soluble guanylyl cyclase (sGC)-cGMP-PKG signaling pathway leading to elevated diastolic stiffness in human HF [32]. From the aspect of the RV, in rat hearts exposed to ischemia and reperfusion injury, unlike the healthy RV, neither ischemic preconditioning nor stimulation of the sGC-cGMP-PKG axis could rescue the hypertrophic and failing RV due to pulmonary trunk banding [38]. Likewise, in human trials on HFrEF patients with pulmonary arterial hypertension boosting the sGC-cGMP-PKG signaling pathway, either sGC stimulation (riociguat) [39] or phosphodiesterase-5 inhibition (sildenafil) [40,41] failed to significantly reduce pulmonary arterial pressures and improve hemodynamics. Based on these results, one could therefore speculate that the pathophysiological role of PKG downregulation is more predominant in the LV as compared to the RV in the post-ischemic failing heart.

Furthermore, PKA phosphorylates titin’s N2Bus and reduces cardiomyocyte passive tension both in rats [42] and humans [43]. Accordingly, in vitro administration of PKA to HF cardiomyocytes corrects high titin-based passive tension to control values, suggesting reduced titin phosphorylation of human biopsies from HF patients [34]. Indeed, we found a biventricular reduced PKA activity and thereby a global passive stiffness lowering effect upon PKA incubation in our HFrEF rats.

Finally, PKC also phosphorylates titin’s PEVK element and increases titin-based passive tension both in health [44] and disease [45]. *Belin* et al. repeatedly showed augmented PKCα–induced myofilament protein phosphorylation contributing to myofilament dysfunction in post-ischemic HF rats [46,47]. Accordingly, in our HFrEF rats, we also showed a biventricular increased PKC activity with no significant correction of in vitro PKCα administration on cardiomyocyte stiffness, suggesting already saturated PKC-targeted phosphosites on titin. According to our data, titin phosphorylation by different kinases can produce different mechanical effects apparently depending on where the kinase phosphorylates the titin spring [20], but also distinct effects on both ventricles.

These results are in agreement with previous experimental reports showing no impact of PKCα treatment on active force generation and Ca^2+^-sensitivity in failing LV cells likely due to increased activity and signaling of PKCα [46]. However, in HFrEF rats, here we observed interventricular differences in the Ca^2+^-sensitivity of the contractile machinery and the phosphorylation pattern of myofilament proteins involved in the regulation of Ca^2+^-sensitivity. Our findings are clearly in line with earlier observations demonstrating higher Ca^2+^-sensitivity of post-MI LV cardiomyocytes than that of HF RV cells, while Ca^2+^-sensitivity was not changed in the HFrEF RV as compared to either Sham-operated animals here or unoperated age-matched controls earlier [47]. Among myofilament proteins, cTnI phosphorylation plays a complex but direct role in the regulation of Ca^2+^-sensitivity of force production, especially during the progression of HF [48]. Accordingly, we found a hypophosphorylated cTnI both in the RV and LV of HFrEF rats. In addition, site-specific Ser-23/24 phosphorylation of cTnI also showed a biventricular reduction in the HFrEF group, although Ca^2+^-sensitivity of force production was increased only in the LV. In female CD1 mice, following LV MI, increased LV cardiomyocyte Ca^2+^-sensitivity was associated with myofilament protein oxidation (e.g., Tm) and increased S-glutathionylation (e.g., titin) as well as hypophosphorylation of cTnI and cMLC-2 [49]. In our previous work on female OF-1 mice following anterior MI, we demonstrated regional LV differences in myofilament protein function, phosphorylation and oxidation. Namely, Ca^2+-^sensitivity of force production and level of cTnI phosphorylation were lower, while myofilament protein SH oxidation and carbonylation levels were higher in the MI anterior wall than equally the controls or the remote areas of the LV [50]. Moreover, earlier in a porcine model of pacing-induced HF, we demonstrated a high cell-to-cell variability of PKA-dependent cTnI phosphorylation and Ca^2+^-sensitivity of force production at the LV pacing site [51]. Nonetheless, in a recent study, unaltered RV cardiomyocyte Ca^2+^-sensitivity might be attributable to the complex regulatory effect of hypophosphorylation of cMLC-2 [52], cTnI, and cMyBP-C in HFrEF animals. On the other hand, we observed a striking difference in cMyBP-C Ser-282 site-specific phosphorylation in the RV vs. LV in HFrEF rats. *Sadayappan* et al. confirmed a unique regulatory role of cMyBP-C Ser-282 phosphorylation presumably by CaMKII for the subsequent phosphorylation of other sites in maintaining sarcomere organization and function [53]. Hypophosphorylated Ser-282 of cMyBP-C could be explained by the significant reduction of CaMKII activity in the RV of our HFrEF rats. In addition, cMyBP-C phosphorylation was shown to be cardioprotective and help protect the myocardium from ischemic injury [54]. Recent clinical and experimental data also suggest that cMyBP-C phosphorylation modulates the relative cross-bridge detachment rate with respect to attachment rate and thereby mediates diastolic function [55]. Previously, we provided evidence on the direct impact of oxidate stress on CaMKII-mediated myofilament protein phosphorylation and function [56], of which this interaction is likely important in the post-ischemic heart as well. Finally, upstream signaling mechanisms might also show heterogeneity, since interventricular differences were reported in inotropic responses to α_1_-adrenergic stimulation in control mice [57], as well as in adenylyl cyclase activities in LV MI-induced HF rats [58].

Beyond diverse signaling alterations in two ventricles of the failing heart, we did not observe significant changes in the RV and LV morphological parameters, namely cardiomyocyte size and collagen gene expression, of our moderate-stage HFrEF rats. In male rats after LV MI biventricular dilation, hypertrophy and fibrosis developed in failing animals, but only LV dilation and RV fibrosis were observed in post-MI non-failing animals [59]. In failing post-ischemic rats, LV fibrosis was associated with higher gene expression levels of collagen I and TGF-β, unlike the RV, in which gene expression levels of collagen I and TGF-β did not change at all [59]. Similar time-dependent alteration of myocardial proteome during HF development was reported in mice following TAC (transverse aortic constriction) [60]. In particular, small heat shock proteins (sHSP) were accumulated in the LV, but remained unaffected in RV for 6 weeks, suggesting both a delayed onset of LV backward failure and a poor capacity of RV oxidative defense [12,13,14,60]. In addition, in vivo data from another TAC model proposed an important role for Tm phosphorylation against cardiac stress and suggested that Tm de-phosphorylation leads to compensated but insufficient hypertrophy against cardiac stress [61]. These observations are again in line with ours, showing here higher LV vs. RV Tm phosphorylation in the HFrEF group. Indeed, in male mice that have undergone TAC, interventricular differences were described in cardiac remodeling [62]. However, diastolic dysfunction appears as a dynamic spectrum of impairments when RV dysfunction develops secondary to LV dysfunction in these animals. Collectively, complex and distinct remodeling processes supposedly work to prevent RV distention that impairs LV performance through ventricular interdependence [9]. As a matter of fact, elevated RV titin-based stiffness was reported in human patients with primary pulmonary arterial hypertension as well [63], but its mechanism seems to be different than that of the post-ischemic failing heart in our study, since only PKA could lower RV cardiomyocyte stiffness, but neither CaMKIIδ nor PKCα incubation had significant mechanical effect in those experiments [64].

As a matter of fact, RV dysfunction is present in HF with preserved LV EF (HFpEF) as well [2]. Although comorbidities of HFpEF trigger oxidative stress and inflammation [32], the pathomechanism of HFpEF is less understood than that of HFrEF. HFpEF is a complex HF phenotype that might also involve interventricular differences of cardiomyocyte signaling pathways under oxidative conditions as well, partly leading to the failure of clinical studies so far. Still, this theory needs to be proven by future experiments.

## 5. Conclusions

In summary, distinct signaling pathways mediate the phosphorylation of sarcomeric proteins upon high oxidative stress in the RV and LV of the post-ischemic failing rat heart. These findings may change the concept of treatment, as the RV and LV differ in their intracellular signaling and thus show differential modulation of proteins at certain diseases (Table 1). Our data fill a critical void by elucidating mechanistic insights involved in HFrEF RV and LV remodeling and dysfunction, which is necessary to improve pathophysiological understanding and inform the development of strategies for treatment and prevention. Thus, our results may open a new avenue to selectively target the RV independent from the LV and vice versa.

## Figures and Tables

**Table 1:** Summary of interventricular differences of the post-ischemic heart.

	RV	LV
Molecular basic structure	different from LV	different from RV
Post-MI ischemic stress	no	yes
Transmyocardial scar formation	no	yes
Volume overload	yes	yes
Consequence of systemic humoral and inflammatory activation	yes	yes
Regulation of signaling pathways		
*CaMKII*	down	up
*PKG*	down	down
*PKA*	down	down
*PKC*	up	up
Cardiomyocyte passive stiffness	increased	increased
Cardiomyocyte Ca^2+^-sensitivity	unaltered	increased

## Data Availability

Not applicable.
